# Supplementary material for: Patterns of Intron Gain and Loss in Fungi
Source: PLoS Biol. 2004 Nov 30;2(12):e422. doi: 10.1371/journal.pbio.0020422 (PMC532390; doi:10.1371/journal.pbio.0020422)
Supplement: Table S1 — Also available at http://genes.mit.edu/NielsenEtAl/. (4.3 MB ZIP). [file pbio.0020422.st001.zip › NielsenEtAl/html/997.html]

AN2762.1.NCU02291.1.MG06561.1.FG10804.1


```
 CLUSTAL W (1.82) Multiple Sequence Alignments - Introns Inserted


Sequence 1: MG06561.1	500 aa
Sequence 2: FG10804.1	426 aa
Sequence 3: AN2762.1	424 aa
Sequence 4: NCU02291.1	436 aa
Alignment Length: 513 aa
Number Identitical Residues: 258 aa
Alignment Score (without introns) 11672


MG06561.1 	MSR--------YLTRRLAQTTAS-LRLSSGRRCYSAK---ASFDWKDPLNSKSLLTEEEV
NCU02291.1	MSRILARRCPQGLLRQAFSPSRTNVTTTPVRAFASATSNAPSFNWEDPLNHKSLLTEDEI
FG10804.1 	MFR--------PILRQCAQRAATPLRPCVTRTFASASG--PSFNWEDPLASNNLLTEEER
AN2762.1  	MN-------PSSFFRTASR-SAGRLSKIP----AASRGYATAFKWEDPLLASELYTEDEL
          	*        .. : *     :   :         :: . :.:*.*:***  ..* **:* 

MG06561.1 	AISETAERYCQEKLAPRVLG~TLRVRFLDLVIVSR2AILSEMGELGLLGATISGHGCAGV
NCU02291.1	AIGETAERYCQEQLFPRVLQ~AYRDEHYDP-----~KILEEMGDLGLLGATIEGYGCAGV
FG10804.1 	AIGDTAERYCQEQLLPRVLK1AYRDESYDP-----~KILEEMGELGLLGATIEGYDCAGA
AN2762.1  	AIQETARQYCQERLQPRVLD1AYRNENYDR-----~KILEEMGELGLLGASIEGYGCAGV
          	** :**.:****:* ****  : * .  *        **.***:******:*.*:.***.

MG06561.1 	STVAGALITRAVERVDSGYRSGMSVQSSLVMGGIDYWGSTEQKERFLPQMAAGKLLGAFG
NCU02291.1	SSVASGLITRAVERVDSGYRSGMSVQSSLVMGPINDFGTAEQKERFLPGMAKGKIIGAFG
FG10804.1 	SSVAGGLITRAVERVDSGYRSGMSVQSSLVMGGIYEHGSAEQKEKFLPKMAKGKMLGAFG
AN2762.1  	STVASGLITKEVERVDSGYRSGMSVQSSLAMTAIHEFGTQELKDRLLPELAKGKLAGCFG
          	*:**..***: ******************.*  *   *: * *:::** :* **: *.**

MG06561.1 	LTEPNHGSDPASMETVARPHPTKKGFYSLSGSKTWITNSPIADILLVWAKLADGEGPSKI
NCU02291.1	LTEPNHGSDPGSMETVARPHPTKEGVYLLSGSKTWITNSPIADVLVVWAKLQETG---KI
FG10804.1 	LTEPNHGSDPGSMETVAKPHPTKKGYYSLSGAKTWITNSPIADVLMVWAKLQETG---KI
AN2762.1  	LTEPNHGSDPGSMETIAREHPTKKGVYLLSGSKTWITNSPISDILIVWAKLQSTG---KI
          	**********.****:*: ****:* * ***:*********:*:*:***** .     **

MG06561.1 	RGFLVERKNCPPGTLETPAIKNKTGLRASLTGMIHLDNCPIPEENMFPTVTGLKGPFTCL
NCU02291.1	RGFLVERSACPPGTLETPQIKNKTGLRASITGMIQLDNCPVPKENMFPEIEGLKGPFTCL
FG10804.1 	RGFLVERKDCPPGTLETPAIKDKNGLRASITGMIQLDNCPVPENNMFPEVEGLRGPFSCL
AN2762.1  	RGFVVERSKCPPGTLETPALKNKSALRASITGMIQLDDCPVPVENMFPDVEGLTGPFTCL
          	***:***. ********* :*:*..****:****:**:**:* :**** : ** ***:**

MG06561.1 	NSARFGIAMGVMGALEDCIAATREYALERSQF-GRPLASFQLVQKKLADAATDAAYGSLA
NCU02291.1	NSARFGIAFGMMGALEDCLARARTYALERKQFKGNPLAKYQLVQKKLAEAATDVAYGTLA
FG10804.1 	NNARYGIAMGVMGALEDCLARARTYSLERKQFKGNPLARYQLVQKKLADASTDAAYGTLA
AN2762.1  	NSARLGIAFGAMGALEDCIDRARTYALERKQFKGNPLAKYQLIQKKLADAVTDATYGTLA
          	*.** ***:* *******:  :* *:***.**.*.*** :**:*****:* **.:**:**

MG06561.1 	AIQVGRLIDEGKSVPDMISMVKRENCDRALRNART~LQEVLGGNAVSDEYRIGRHVANLF
NCU02291.1	AIQVGRLKDEGKATPEMISMIKRQNCDRTLYHART~LQEIFGGNAVSDEYHIGRVVANLY
FG10804.1 	AIQVGRLKDEGKATPEMISMVKRQNCDTALRNARL~LQEIFGGNAASDEYMIGRHVANLF
AN2762.1  	ATQVARLKDAGKCTPEMISLVKRQNCDRALANSRT2LQEVFGGNAASDEYHIARHVANLF
          	* **.** * **..*:***::**:*** :* ::*  ***::****.**** *.* ****:

MG06561.1 	VTQTYEGQSDIHS1LILGRAVTGIQA2LLRWVVIGDGSLASFVFHRDVGSESPLRVKPHA
NCU02291.1	VTQTYEGQSDIHS1LILGRAITGVQA~FV-------------------------------
FG10804.1 	VTQTYEGQSDIHS1LILGRAITGIQA~FV-------------------------------
AN2762.1  	VVQTYEGQSDIHA1LILGRAITGVQA~FV-------------------------------
          	*.**********: ******:**:** ::                               

MG06561.1 	QPYCQ1FYYVGDCRALHLTKKE1MCFAQSRQRVAQPPQLG
NCU02291.1	-----~----------------~-----------------
FG10804.1 	-----~----------------~-----------------
AN2762.1  	-----~----------------~-----------------
          	
```
